# Health Status and Driving Among Community-Dwelling Older Adults

**DOI:** 10.3390/healthcare13222866

**Published:** 2025-11-11

**Authors:** Seoyoung Park, Se-Won Kang

**Affiliations:** 1 Department of Nursing, Gyeongsang National University, 861-15 Jinju-daero, Jinju-si 52727, Republic of Korea; masternur@gnu.ac.kr; 2 Department of Nursing, Dongseo University, 47 Jurye-ro, Sasang-gu, Busan 47011, Republic of Korea

**Keywords:** health, driving, community, older adults

## Abstract

**Background/Objectives:** Maintaining independent mobility among older adults requires complex cognitive and physical health and is influenced by various health-related factors. This study sought to examine the relationship between health-related factors and driving among community-dwelling older adults by comparing the health status of currently driving individuals and those who have ceased driving. **Methods:** A secondary data analysis was conducted using the 2023 Korean Elderly Survey, collected between 4 September and 12 November 2023. A total of 4114 individuals aged 65 years or older were included. Statistical analyses were performed using chi-square tests, independent t-tests, and weighted binary logistic regressions via IBM SPSS for Windows. **Results:** Significant health-related factors for driving cessation included having ≥2 chronic diseases (OR = 1.22, *p* = 0.041), diagnosed depression (OR = 3.64, *p* = 0.030), Instrumental Activities of Daily Living dependency (OR = 1.67, *p* = 0.001), visual discomfort (OR = 1.18, *p* = 0.048), depression risk (OR = 1.34, *p* = 0.015), suspected cognitive impairment (OR = 1.73, *p* < 0.001), and poor self-rated health (OR = 1.21, *p* = 0.029). None of the participants with Parkinson’s were currently driving, whereas polypharmacy (≥5 medications) was not statistically significant (OR = 0.77, *p* = 0.222). Chronic diseases that may affect driving were also not statistically significant. **Conclusions:** This study highlights the fact that older drivers may have difficulty recognizing health-related risks that affect driving. To support safe mobility, it is essential to implement a health-centered assessment of driving fitness, including an appropriate evaluation cycle, and promote continuous education to raise awareness among older adults.

## 1. Introduction

The high proportion of older adults in the population and their health status are closely linked not only to individual quality of life but also to broader societal burdens. Many countries worldwide are experiencing an increase in the proportion of older individuals, and South Korea entered a super-aged society in 2025, with 20.3% of its population aged 65 years and older. This figure is projected to increase to 25.3% by 2030 and 34.3% by 2040 [1]. Generally, older adults face various health challenges, including a decline in physical function, cognitive deterioration, and an increase in chronic diseases, all of which affect their ability to perform daily activities. Aging gradually affects everyday decision-making and reaction speed, necessitating increased societal attention [2]. Therefore, accurately assessing the health status of older adults and understanding its relationship with functional capacity have emerged as critical issues [3].

Among various indicators of autonomy and functional independence, the ability to drive is often considered a reflection of an individual’s physical and cognitive health [4]. In South Korea, the number of older individuals holding a driver’s license increased from 2.29 million in 2015 to 5.17 million in 2024, accounting for 52% of the population aged 65 years and older. This number is projected to reach approximately 9.83 million by 2050 [5]. The proportion of older drivers among all licensed drivers has also risen steadily from 7.6% in 2015 to 14.9% in 2024 [6].

Driving requires complex cognitive and physical skills, and age-related health declines can significantly affect driving ability [7]. Older individuals may experience difficulties in recognizing traffic signals, making decisions at intersections, adjusting driving speeds, and responding promptly, which can make them more vulnerable to specific types of traffic collisions [8]. Moreover, the number of traffic accidents per 10,000 licensed drivers aged 65 years and older has been increasing, with the fatality rate among older drivers rising from 13.3% in 2012 to 24.3% in 2021 [9]. In response, some countries have mandated regular health checkups or driving aptitude assessments for drivers older than a certain age. However, driving cessation still largely relies on self-assessment in many cases [10]. For this reason, self-report assessment tools addressing driving risk in older adults have been developed and are currently in use [11,12].

Health status plays a pivotal role in driving decisions among older adults. Studies have shown that older individuals who continue to drive tend to be physically and mentally healthier. Driving supports independent living and mobility, which in turn promotes social participation and autonomy, potentially leading to reduced depressive symptoms and increased life satisfaction [13,14]. According to previous studies, older adults who are currently driving report significantly better subjective health, fewer depressive symptoms, and relatively superior cognitive function than non-drivers and those who have ceased driving [15,16]. Comparative analyses between drivers and nondrivers have also shown that drivers maintain better overall cognitive and physical functions [17]. In particular, driving ability in older adults is closely associated not only with basic visual acuity and reflexes but also with cognitive functions such as memory, attention, and judgment, as well as physical capabilities such as motor speed and muscle strength [18]. However, despite experiencing a decline in vision, hearing, reaction time, and judgment, many older drivers tend to underestimate the impact of health issues on driving fitness [19].

While public discourse surrounding older drivers has often focused on accident rates and safety concerns, more fundamental questions remain underexplored—such as whether older adults possess adequate health conditions for safe driving and what specific health factors influence their decision to cease driving [20]. Driving cessation is not merely a loss of transportation; it can lead to diminished autonomy and weakened social connections, resulting in increased depression, loneliness, and reduced life satisfaction. Despite these implications, research on changes in quality of life following driving cessation remains insufficient, and there is a pressing need to explore how health status influences mobility transitions in later life.

In contrast, the criteria for assessing driving fitness in older adults remain inconsistent and often rely on the subjective judgment of healthcare professionals or family members. Therefore, an objective and standardized evaluation system for determining driving eligibility is urgently required.

Social perceptions of older drivers must also shift toward a more balanced approach that considers both safety and individual rights. This study was aimed at enhancing our understanding of how health-related factors contribute to mobility decisions and identifying other factors to be considered in routine geriatric care by analyzing the relationship between the health status of older adults and driving.

### Study Purpose

We aimed to investigate the relationship between health-related factors and driving among community-dwelling older adults.

1. To compare the general characteristics between currently driving individuals and those who have ceased driving.
2. To examine differences in health status between driving individuals and those who have ceased driving.
3. To identify health-related factors associated with driving cessation among older adults.

## 2. Materials and Methods

### 2.1. Study Design

We investigated the relationship between health-related factors and driving among community-dwelling older adults through descriptive research based on secondary data analysis utilizing raw data from the 2023 Korean Elderly Survey [21].

### 2.2. Study Participants

Of the 10078 individuals who participated in the survey, 4114 were selected for this study based on the following criteria: (1) being aged 65 years or older, (2) having responded to the survey independently without assistance from children or others, (3) being a current or prior driver, and (4) having no missing data on key variables.

### 2.3. Data Collection

The raw data used in this study were obtained from the 2023 Korean Elderly Survey conducted by the Ministry of Health and Welfare and the Korea Institute for Health and Social Affairs. The dataset was accessed and analyzed according to the data usage procedures outlined on the MicroData Integrated Service (MDIS) website of the National Statistical Office (https://mdis.kostat.go.kr/eng/index.do, accessed on 27 May 2025).

The Korean Elderly Survey is conducted every 3 years under the Elderly Welfare Act to assess the overall living conditions and needs of older adults residing in the community. It serves as foundational data for improving the quality of life of older adults and establishing welfare policies.

The 2023 survey was conducted by 176 trained interviewers between 4 September and 12 November 2023. Interviewers visited all households within the preselected sampling areas and conducted one-on-one, face-to-face interviews with older individuals residing in those households. The survey employed the tablet PC-assisted personal interview (TAPI) method, in which interviewers used tablet PCs programmed with survey content to collect responses. In total, 10078 individuals completed the survey.

Raw data were collected using a three-stage stratified cluster sampling method. In the first stage, stratification was based on the regional distribution across 17 metropolitan and provincial areas. The second stage involved the stratification of each region into urban (dong) and rural (eup/myeon) areas. In the third stage, sampling was further stratified according to the characteristics of the survey districts (e.g., apartment vs. general housing) within each region and area type.

### 2.4. Study Variables

#### 2.4.1. Driving

Driving was defined as operating a vehicle independently. Driving cessation was defined as being a driver in the past but no longer driving. It was assessed using a single survey item: “Are you driving now?” Participants were asked to choose one of the following responses: (1) “Yes, I’m driving” (classified as the driving group), (2) “No, I used to drive, but I don’t anymore” (classified as the driving cessation group), or (3) “I’ve never driven.” Individuals who reported never having driven were excluded from this study.

#### 2.4.2. Health Status

Health status variables included factors potentially associated with driving behavior, such as (1) diagnosed chronic diseases; (2) number of medications; (3) daily life performance; (4) discomfort in vision, hearing, and mobility; (5) risk of depression; (6) cognitive impairment; (7) sleep quality; and (8) self-rated health.

(1) **Diagnosed chronic diseases**

This variable comprised both the total number of chronic conditions diagnosed by a physician and the presence of 14 specific chronic diseases commonly observed among older adults, and is known to potentially affect driving ability [22]. These conditions include cerebral infarction, heart disease (angina, myocardial infarction, and heart failure), diabetes mellitus, arthritis, depression, dementia, Parkinson’s disease, insomnia, glaucoma, presbycusis, cancer, urinary incontinence, and anemia. The “others” category included conditions such as gout and panic disorder.

(2) **Number of medications**

This variable referred to the number of different types of medications currently being taken by the participant as prescribed by a physician.

(3) **Daily life performance**

Daily life performance was assessed using two standardized measures: Activities of Daily Living (ADL) and Instrumental Activities of Daily Living (IADL) [23]. Participants were asked to indicate the level of assistance they required for each item over the past week, with response options being “completely independent,” “partial assistance,” and “complete assistance.” ADL included seven items: dressing, washing/brushing teeth, washing hair, bathing/showering, eating prepared meals, getting up and moving outside the room, using the toilet, managing hygiene after elimination, and controlling urination/defecation. The IADL included ten items: grooming, household chores, meal preparation, laundry, shopping/financial transactions, making and receiving phone calls, taking medications, managing finances, going out nearby, and using transportation. Participants were classified as “independent” if they reported complete independence in all items. If they required partial or complete assistance in even one item, they were classified as “dependent.”

(4) **Discomfort in vision, hearing, and mobility**

Discomfort was assessed in three domains: vision, hearing, and mobility. Participants responded to the question, “To what extent do you experience discomfort in daily life due to vision, hearing, or mobility?” Each domain was rated on a three-point scale: “very uncomfortable,” “somewhat uncomfortable,” and “not uncomfortable.” For analysis, responses were dichotomized into “uncomfortable” (including “very uncomfortable” and “somewhat uncomfortable”) and “not uncomfortable.” Vision referred to activities, such as watching television and reading newspapers. Hearing included making phone calls or conversing with others. Mobility referred to movement both inside and outside the home.

(5) **Risk of depression**

Depression was measured using the Korean version of the Short Form of the Geriatric Depression Scale (SGDS-K), a widely used screening tool for identifying depressive symptoms in older adults. The SGDS-K is a shortened version of the original Geriatric Depression Scale (GDS) adapted for the Korean population [24].

The scale consists of 15 items with binary response options (“yes” or “no”). Each ‘yes’ response was scored as 1 point, and the total score was calculated by summing the points. A total score greater than 5 indicated a risk of depression. At the time of its development, the SGDS-K demonstrated high internal consistency with a Cronbach’s alpha of 0.88. In the present study, the reliability of the tool was confirmed, with a Cronbach’s alpha of 0.825. Participants with scores above 5 were classified as being at risk for depression.

(6) **Cognitive Impairment**

Cognitive impairment was assessed using the Korean version of the Mini-Mental State Examination (MMSE-K), a validated screening tool adapted from the original Folstein MMSE for Korean populations [25]. The MMSE-K evaluates the cognitive function across various domains, with scores ranging from 0 to 30.

Scores were expressed as the means ± standard deviations (S.D.), and cutoff points for potential cognitive impairment were determined based on deviations from the mean. Specifically, scores falling below –1.0 S.D., –1.5 S.D., and –2.0 S.D. have been suggested as thresholds for identifying possible cognitive decline [26]. In clinical practice, individuals scoring below –1.5 S.D. are typically considered for further diagnostic evaluation [27]. In this study, age- and education-adjusted participants with MMSE-K scores below –1.5 S.D. were classified as being at risk for cognitive impairment.

(7) **Quality of Sleep**

Sleep quality was assessed using a single-item measure rated on a five-point Likert scale ranging from 1 (very poor) to 5 (very good or excellent). Participants were asked, “How would you rate your overall quality of sleep these days?”

Although depression, dementia, and insomnia were included among the chronic diseases diagnosed in this study, symptoms such as depressive mood, cognitive decline, and poor sleep quality are often regarded as part of the normal aging process rather than as distinct medical conditions. Consequently, individuals may experience these problems without receiving a formal diagnosis from their physician. Therefore, these constructs were included as separate variables in the analysis to capture potential concerns that may not be reflected in clinical diagnoses.

(8) **Self-Rated Health**

Self-rated health (SRH) refers to an individual’s subjective assessment of their overall health status. In this study, SRH was measured using a single-item question: “How would you rate your overall health status these days?” Responses were recorded on a five-point Likert scale ranging from 1 (very poor) to 5 (very good/excellent).

#### 2.4.3. Adjusted Variables

The general characteristics of the study participants included sex, age, educational level, household income quintile, employment status, and living arrangements.

- Age was categorized into five-year intervals, starting from 65 years and above.
- Educational level was classified into four groups: elementary school or lower, middle school, high school, and college or higher.
- Household income was assessed as the annual household income and divided into five quintiles, each representing 20% intervals.
- Employment status was determined based on whether the participant was currently engaged in income-generating work and was categorized as either employed or unemployed.
- Living arrangements were divided into three categories: living alone, living with a spouse only, and living with other family members or individuals.

### 2.5. Ethical Considerations

This study used data from the 2023 National Survey of Older Koreans approved by the Institutional Review Board (IRB) of the Korea Institute for Health and Social Affairs (KIHASA) (IRB No. 2023-078, approved on 28 July 2023). The survey was administered by trained interviewers who first obtained written informed consent from the participants before conducting the questionnaire. Researchers wishing to access the dataset may use the MDIS provided by Statistics Korea. After a brief approval process was completed, anonymized data were made available. The dataset did not contain any personally identifiable information, ensuring that individual respondents could not be identified.

### 2.6. Data Analysis

Data were analyzed using IBM SPSS Statistics (version 23.0; IBM, Armonk, NY, USA).

(1) Descriptive statistics, including frequencies, percentages, means, and standard deviations, were used to summarize the participants’ general characteristics and health status.
(2) Differences in the current driving status according to general characteristics and health status were examined using the chi-square test and independent sample t-test.
(3) To identify the health-related factors associated with driving cessation among older adults, a weighted binary logistic regression analysis was conducted.
(4) The level of statistical significance was set at *p* < 0.05.

## 3. Results

### 3.1. Differences Between the Driving and Driving Cessation Groups According to General Characteristics

Among the 4114 participants, 1735 (42.2%) had discontinued driving, while 2379 (57.8%) were currently driving. The differences in the general characteristics between the two groups are presented in Table 1.

Age was the most significant differentiating factor between the groups (χ^2^ = 798.68, *p* < 0.001). The overall mean age of the participants was 72.6 ± 5.38 years. The driving cessation group had a significantly higher mean age (75.3 ± 6.23 years) than the driving group (69.9 ± 4.53 years), with a statistically significant difference (t = 30.54, *p* < 0.001). Most variables showed statistically significant differences between the two groups: employment status (χ^2^ = 461.38, *p* < 0.001), household income quintile (χ^2^ = 362.69, *p* < 0.001), educational level (χ^2^ = 177.88, *p* < 0.001), and living arrangement (χ^2^ = 42.72, *p* < 0.001). By contrast, there was no statistically significant difference between the two groups in terms of sex (t = 2.50, *p* = 0.120).

### 3.2. Differences Between the Driving and Driving Cessation Groups According to Health Status

Health status variables that showed significant differences between the driving and driving cessation groups are listed in Table 2.

The average number of chronic diseases diagnosed among all participants was 1.91 ± 1.42. The driving group had significantly fewer chronic conditions (1.6 ± 1.33) than the driving cessation group (2.2 ± 1.50), with a statistically significant difference (t = 13.72, *p* < 0.001). Similarly, the average number of medications was 1.8 ± 1.49 for all participants, with the driving group averaging 1.5 ± 1.39 and the driving cessation group averaging 2.1 ± 1.58 (t = 11.98, *p* < 0.001). The proportion of participants taking five or more different medications was significantly different between the two groups (χ^2^ = 24.08, *p* < 0.001). Among specific chronic diseases, the largest differences were observed in cerebral infarction (χ^2^ = 46.57, *p* < 0.001), followed by presbycusis (χ^2^ = 36.93, *p* < 0.001), depression (χ^2^ = 35.19, *p* < 0.001), diabetes mellitus (χ^2^ = 31.83, *p* < 0.001), heart disease (χ^2^ = 20.06, *p* < 0.001), arthritis (χ^2^ = 19.40, *p* < 0.001), dementia (χ^2^ = 19.02, *p* < 0.001), insomnia (χ^2^ = 13.33, *p* < 0.001), and urinary incontinence (χ^2^ = 4.55, *p* < 0.001). Notably, none of the participants diagnosed with Parkinson’s disease were currently driving. In contrast, no statistically significant differences were found between the two groups for glaucoma (χ^2^ = 1.09, *p* = 0.336), cancer (χ^2^ = 3.17, *p* = 0.076), anemia (χ^2^ = 0.81, *p* = 0.375), or other driving-related diseases (χ^2^ = 0.25, *p* = 0.711). There were also significant differences between the two groups in ADL (χ^2^ = 163.20, *p* < 0.001), IADL (χ^2^ = 238.29, *p* < 0.001), discomfort related to vision (χ^2^ = 83.53, *p* < 0.001), hearing (χ^2^ = 234.79, *p* < 0.001) or mobility (χ^2^ = 177.01, *p* < 0.001), risk of depression (χ^2^ = 171.25, *p* < 0.001), cognitive impairment (χ^2^ = 65.50, *p* < 0.001), quality of sleep (χ^2^ = 117.56, *p* < 0.001; t = −11.15, *p* < 0.001), and self-rated health (χ^2^ = 248.45, *p* < 0.001; t = −17.80, *p* < 0.001).

As shown in Table 2, some participants who were currently driving presented with multiple chronic conditions, polypharmacy, functional dependency, sensory discomfort, risk of depression, cognitive impairment, poor sleep quality, and low self-rated health.

### 3.3. Probability of Driving Cessation by Health Status

The probability of driving cessation according to participants’ health status is presented in Table 3. Before the analysis, the suitability of the model was verified. The weighted binary regression model was adequate (χ^2^ = 1447.90, *p* < 0.001; Hosmer and Lemeshow χ^2^ = 9.03, *p* = 0.340), and the explanatory power of the model was 39.9%.

In terms of health status, the number of chronic diseases diagnosed was significantly associated with driving cessation. Participants with two or more chronic diseases were 1.22 times more likely to discontinue driving than those with fewer than two conditions (Odds Ratio [OR] = 1.22, *p* = 0.041, 95% CI: 1.008–1.464). Among the 14 chronic diseases examined, only depression and Parkinson’s disease were significantly associated with driving cessation. Individuals diagnosed with depression were 3.64 times more likely to have stopped driving (OR = 3.64, *p* = 0.030, 95% CI: 1.133–11.706). Notably, none of the participants diagnosed with Parkinson’s disease were currently driving. Polypharmacy, defined as the use of five or more different medications, was not a statistically significant predictor of driving cessation. Several functional and psychological health indicators were significantly associated with driving cessation. Participants with dependent IADL status were 1.67 times more likely to have stopped driving (OR = 1.67, *p* = 0.001, 95% CI: 1.243–2.238). Visual discomfort was also a significant factor (OR = 1.18, *p* = 0.048, 95% CI: 1.001–1.396), as was the risk of depression (OR = 1.34, *p* = 0.015, 95% CI: 1.059–1.691). Those with suspected cognitive impairment had 1.73 times higher odds of having discontinued driving (OR = 1.73, *p* < 0.001, 95% CI: 1.422–2.116). Additionally, individuals with poor self-rated health status were 1.21 times more likely to have discontinued driving (OR = 1.21, *p* = 0.029, 95% CI: 1.020–1.445).

According to the findings of this study, currently driving individuals may experience difficulties while operating a vehicle. Therefore, an additional analysis was conducted on the 2379 participants in the driving group to examine the challenges they faced while driving. The participants were allowed to report all applicable difficulties. A total of 638 individuals (28.7%) responded that they experienced difficulties while driving. The reported difficulties included poor vision (31.3%, *n* = 214), reduced sense of speed (24.9%, *n* = 170), misunderstanding of road signs (21.4%, *n* = 146), difficulty moving quickly (18.7%, *n* = 128), poor hearing (3.1%, *n* = 21), and other issues (0.6%, *n* = 4).

## 4. Discussion

This study aimed to identify the health-related factors associated with driving cessation among older adults. Health-related factors were found to be significant.

### 4.1. General Characteristics Associated with Driving Among Older Adults

Age, educational level, household income quintile, employment status, and living arrangements significantly influenced driving cessation among older adults. Specifically, older age was associated with a higher likelihood of driving cessation, whereas lower levels of education, income, and unemployment were linked to increased odds of discontinuing driving. Compared with individuals living alone, those living with only a spouse had a significantly lower likelihood of ceasing to drive.

Compared with the 65–69-year age group, the likelihood of driving cessation increased progressively with age, rising by 2.74, 4.57, 9.36, and up to 14.63 times across the older age brackets. These findings align with those of Schouten et al. (2021) [8], who identified aging as a major factor in driving restrictions and cessation. This suggests that age-related decline in physical and cognitive function may limit the ability to continue driving. Furthermore, individuals with lower educational levels were 1.9 to 2.3 times more likely to stop driving. This result is consistent with the findings of Moon and Park (2020) [2], who studied older adults in Korea and noted that lower education may hinder access to information and health management, thereby negatively affecting driving continuation. Household income quintiles and employment status were significant factors. Compared with higher-income groups, individuals in lower-income groups were 1.03 to 1.54 times more likely to stop driving, while unemployment was associated with up to a 2.45-fold increase in driving cessation. These results suggest that economic constraints and reduced social engagement may adversely affect the ability to continue driving. Supporting this, Dickerson et al. (2024) [20] found that reduced social activity, financial limitations, and unemployment were key factors in driving cessation among older adults in the U.S. and Europe, emphasizing the importance of economic conditions and social networks in maintaining driving ability. In addition, older adults living with a spouse were less likely to cease driving than those living alone. This indicates that spousal interactions and social support positively influence continuous driving. These findings are consistent with those of Ang et al. (2020) [28], who reported that interactions between older couples have a meaningful positive impact on driving continuation.

The general characteristics identified in this study suggest that driving cessation among older adults is not merely a personal choice but is closely tied to their social and economic context. This underscores the need for policy approaches that comprehensively consider various environmental factors when addressing driving behaviors of older adults.

### 4.2. Health-Related Factors Associated with Driving Among Older Adults

Health-related factors associated with driving cessation among older adults include two or more diagnosed chronic diseases, depression, dependency on IADL, visual discomfort, risk of depression, suspected cognitive impairment, and low SRH.

Older adults diagnosed with two or more chronic diseases were 1.22 times more likely to stop driving than those diagnosed with fewer than two conditions. This finding is consistent with those of previous studies [2,29,30] that explored the relationship between chronic illnesses and driving performance. Chronic conditions may directly affect driving ability by causing physical fatigue, side effects of medication, and slower reaction times. The diagnosis of depression and the risk of depression have also emerged as significant factors influencing driving cessation. Individuals diagnosed with depression were 3.64 times more likely to stop driving, and those at risk of depression were 1.34 times more likely than those not at risk. Depression is closely linked to reduced attention, impaired judgment, and diminished motivation, all of which negatively affect tasks requiring sustained concentration, such as driving [31]. Older adults with depression often reduce their social activities and avoid going out, which may be associated with voluntary driving cessation. Older adults with IADL dependency were 1.67 times more likely to cease driving than those who were independent. IADL reflects autonomy and functional independence in daily life and is closely related to higher-order tasks such as driving. Individuals dependent on IADL may struggle with complex decision-making and situational responses, making continued driving difficult. These findings align with those of Knoefel, Hossain, and Hsu (2023) [32], who reported that older adults with reduced IADL function were classified as high risk in driving assessments and were more frequently advised to stop driving. Visual discomfort was also a significant factor, with affected individuals being 1.18 times more likely to stop driving than those without such issues. This suggests that visual discomfort can directly affect the ability to perceive surroundings, judge distances, and recognize signals while driving. These findings are consistent with prior research [33] indicating that age-related vision decline, as well as conditions such as cataracts and glaucoma, can compromise driving safety. Those with suspected cognitive impairment were associated with a 1.73-fold increase in the likelihood of driving cessation compared with those without cognitive impairment. Cognitive functions play a critical role in processing information, making judgments, remembering, and maintaining attention while driving. A decline in driving ability has been reported even in the early stages of mild cognitive impairment [34]. As cognitive impairment is directly linked to increased accident risk, voluntary driving cessation often occurs following recommendations from family members or healthcare providers. Low SRH status was another significant factor. In this study, individuals with low SRH were 1.21 times more likely to cease driving than those with high SRH. This result aligns with a previous study [35] involving 7609 older adults in the U.S., which found that those who rated their health as “poor” or “fair” were significantly more likely to stop driving than those who rated it as “good.” These findings suggest that individuals who perceive themselves as unhealthy may find driving physically and psychologically burdensome, which leads to voluntary cessation.

The health conditions associated with driving cessation among older adults, as identified in this study, suggest that driving ability is influenced not only by physical and cognitive functions but also by psychological stability and autonomy. Therefore, driving cessation in older adults should not be approached solely as a safety issue; rather, it requires personalized assessment and support based on individual health status. Among the 14 chronic conditions examined in this study, only depression and Parkinson’s disease showed statistically significant associations with driving cessation. The remaining 12 chronic conditions and the use of five or more medications did not demonstrate significant correlations. When managing chronic illnesses or overall health in older adults, healthcare providers may overlook the implications for driving ability, and older individuals may continue to drive out of habit without fully recognizing the limitations posed by their health status. In particular, polypharmacy in older adults has the potential for adverse drug effects [36], and healthcare professionals should carefully evaluate the impact of medication and educate older drivers to raise awareness about how their treatment may affect driving performance. Among various types of sensory discomfort, only visual discomfort was significantly associated with driving cessation. Driving requires not only vision but also hearing and musculoskeletal functions, which are essential for responding to emergencies. Despite their importance, these factors were not statistically significant in this study. This may indicate that hearing loss or physical mobility limitations are perceived as less critical in older adults’ decisions to cease driving. Furthermore, the study found that one in three older adults currently driving reported experiencing difficulties while driving. These findings highlight the need to consider these factors when evaluating whether older individuals should continue driving.

### 4.3. Study Limitations

This study analyzed the association between the diagnosis of chronic diseases and driving cessation among older adults; however, statistically significant results were not observed for most conditions. One limitation lies in the use of disease diagnosis labels as variables, which do not account for the severity of the condition or treatment status and more accurately reflect an individual’s health status. The absence of disease severity in the analysis is a notable limitation, especially in cases where medical judgment regarding driving ability depends on the degree of illness. Future studies should incorporate more refined health assessments by designing variables that consider disease severity, treatment progress, and functional impact.

Additionally, because this study was based on secondary data analysis, there were inherent limitations in the range of variables that could be included. Thereby, it is difficult to confirm whether driving cessation was voluntary or involuntary. Being a cross-sectional study, this restricts the ability to infer causal relationships between variables over time. For future research in this field, it is recommended to examine urban/rural differences, access to public transportation, and cultural attitudes toward driving.

## 5. Conclusions

Health plays a vital role in shaping older adults’ mobility, which encompasses more than transportation—it reflects autonomy, social engagement, and functional independence. However, the ability to drive safely is inherently tied to one’s physical, cognitive, and psychological health. The findings of this study suggest that older adults with health conditions unsuitable for driving may not fully recognize their limitations, highlighting the need for objective and health-centered assessments of driving fitness. In this study, a diagnosis of depression was associated with a 3.5 times higher likelihood of participants stopping driving, while the increase in odds for other health-related factors was either small or statistically insignificant. Healthcare professionals play a critical role in identifying at-risk individuals and guiding mobility-related decisions. Periodic multidimensional evaluations that incorporate medical assessments, functional screenings, and cognitive testing should be integrated into routine geriatric care. Public education and awareness campaigns can help older adults better understand the impact of health on driving ability and promote informed decision-making. Ultimately, recognizing the loss of driving ability as a health-related transition rather than solely a safety issue allows for more comprehensive and compassionate approaches to aging and mobility.

## Figures and Tables

**Table 1:** Differences between the driving and driving cessation groups according to general characteristics.

Variable		Total (*n* = 4114)	Driving Group (*n* = 2379)	Driving Cessation Group (*n* = 1735)	χ^2^ or t(*p*)
		*n* (%) or Mean ± SD			
Sex	Male	3064 (74.5)	1750 (73.6)	1314 (75.7)	2.50 (0.120)
	Female	1050 (25.5)	629 (26.4)	421 (24.3)	
Age (years)	65–69	1734 (42.2)	1381 (58.0)	353 (20.3)	798.68 (<0.001) 30.54 (<0.001)
	70–74	1116 (27.1)	619 (26.0)	497 (28.6)	
	75–79	702 (17.1)	273 (11.5)	429 (24.7)	
	80–84	407 (9.9)	88 (3.7)	319 (18.5)	
	≥85	155 (3.8)	18 (0.8)	137 (7.9)	
	mean ± SD	72.6 ± 5.38	69.9 ± 4.53	75.3 ± 6.23	
Educational level	≤Elementary school	864 (21.0)	365 (15.3)	499 (28.8)	177.88 (<0.001)
	Middle school	959 (23.3)	494 (20.8)	465 (26.8)	
	High school	1771 (43.1)	1147 (48.2)	624 (36.0)	
	≥College	520 (12.6)	373 (15.7)	147 (8.5)	
Household income quintile *	Lowest 20%	462 (11.2)	175 (7.4)	287 (16.5)	362.69 (<0.001)
	Second 20%	619 (15.1)	225 (9.5)	394 (22.7)	
	Third 20%	875 (21.3)	457 (19.2)	418 (24.1)	
	Fourth 20%	1058 (25.7)	700 (29.4)	358 (20.6)	
	Highest 20%	1100 (26.7)	822 (34.6)	278 (16.0)	
Employment status	Employed	2011 (48.9)	1503 (63.2)	508 (29.3)	461.38 (<0.001)
	Unemployed	2103 (51.1)	876 (36.8)	1227 (70.7)	
Living arrangement	Alone	902 (21.9)	437 (18.4)	465 (26.8)	42.72 (<0.001)
	With a spouse only	2806 (68.2)	1687 (70.9)	1119 (64.5)	
	With family members or others	406 (9.9)	255 (10.7)	151 (8.7)	

* Annual household income was categorized into five quintiles. SD = standard deviation.

**Table 2:** Differences between the driving and the driving cessation groups according to health status.

Variable		Total (*n* = 4114)	Driving Group (*n* = 2379)	Driving Cessation Group (*n* = 1735)	χ^2^ or t(*p*)
		*n* (%) or Mean ± SD			
Number of diagnosed chronic diseases		1.9 ± 1.42	1.6 ± 1.33	2.2 ± 1.50	13.72 (<0.001)
Number of medications	4 or fewer	3952 (96.1)	2316 (97.4)	1636 (94.3)	24.80 (<0.001)
	5 or more	162 (3.9)	63 (2.6)	99 (5.7)	
	Mean ± SD	1.8 ± 1.49	1.5 ± 1.39	2.1 ± 1.58	11.98 (<0.001)
Diagnosed chronic disease					
Cerebral infarction	No	3978 (96.7)	2339 (98.3)	1639 (94.5)	46.57
	Yes	136 (3.3)	40 (1.7)	96 (5.5)	(<0.001)
Heart disease	No	3707 (90.1)	2186 (91.9)	1521 (87.7)	20.06
	Yes	407 (9.9)	193 (8.1)	214 (12.3)	(<0.001)
Diabetes mellitus	No	3014 (73.3)	1822 (76.6)	1192 (68.7)	31.83
	Yes	1100 (26.7)	557 (23.4)	543 (31.3)	(<0.001)
Arthritis	No	3763 (91.5)	2215 (93.1)	1548 (89.2)	19.40
	Yes	351 (8.5)	164 (6.9)	187 (10.8)	(<0.001)
Depression	No	4076 (99.1)	2375 (99.8)	1701 (98.0)	35.19
	Yes	38 (0.9)	4 (0.2)	34 (2.0)	(<0.001)
Dementia	No	4091 (99.4)	2376 (99.9)	1715 (98.8)	19.02
	Yes	23 (0.6)	3 (0.1)	20 (1.2)	(<0.001)
Parkinson’s disease	No	4095 (99.5)	2379 (100)	1716 (98.9)	26.17
	Yes	19 (0.5)	0 (0%)	19 (1.1)	(<0.001)
Insomnia	No	4012 (97.5)	2338 (98.3)	1674 (96.5)	13.33
	Yes	102 (2.5)	41 (1.7)	61 (3.5)	(<0.001)
Glaucoma	No	4059 (98.7)	2351 (98.8)	1708 (98.4)	1.09
	Yes	55 (1.3)	28 (1.2)	27 (1.6)	(0.336)
Presbycusis	No	3994 (97.1)	2342 (98.4)	1652 (95.2)	36.93
	Yes	120 (2.9)	37 (1.6)	83(4.8)	(<0.001)
Cancer	No	4018 (97.7)	2332 (98.0)	1686 (97.2)	3.17
	Yes	96 (2.3)	47 (2.0)	49 (2.8)	(0.076)
Urinary incontinence	No	4073 (99.0)	2362 (99.3)	1711 (98.6)	4.55
	Yes	41 (1.0)	17 (0.7)	24 (1.4)	(0.039)
Anemia	No	4082 (99.2)	2363 (99.3)	1719 (99.1)	0.81
	Yes	32 (0.8)	16 (0.7)	16 (0.9)	(0.375)
Others	No	4084 (99.3)	2363 (99.3)	1721 (99.2)	0.25 (0.711)
	Yes	30 (0.7)	16 (0.7)	14 (0.8)	
Daily life performance					
ADLs	Independent	3905 (94.9)	2347 (98.7)	1558 (89.8)	163.20
	Dependent	209 (5.1)	32 (1.3)	177 (10.2)	(<0.001)
IADLs	Independent	3599 (87.5)	2243 (94.3)	1356 (78.2)	238.29
	Dependent	515 (12.5)	136 (5.7)	379 (21.8)	(<0.001)
Discomfort in vision, hearing, and mobility					
Vision	Not	2641 (64.2)	1666 (70.0)	975 (56.2)	83.53
	Discomfort	1473 (35.8)	713 (30.0)	760 (43.8)	(<0.001)
Hearing	Not	3436 (83.5)	2167 (91.1)	1269 (73.1)	234.79
	Discomfort	678 (16.5)	212 (8.9)	466 (26.9)	(<0.001)
Mobility	Not	3773 (91.7)	2298 (96.6)	1475 (85.0)	177.01
	Discomfort	341 (8.3)	81 (3.4)	260 (15.0)	(<0.001)
Risk of depression	Not at risk	3482 (84.6)	2163 (90.9)	1319 (76.0)	171.25 (<0.001)
	At risk	632 (15.4)	216 (9.1)	416(24.0)	
Cognitive impairment	Not suspected	3354 (81.5)	2039 (85.7)	1315(75.8)	65.50
	Suspected	760 (18.5)	340 (14.3)	420 (24.2)	(<0.001)
Quality of sleep	4 or more	2382 (57.9)	1547 (65.0)	835 (48.1)	117.56
	3 or fewer	1732 (42.1)	832 (35.0)	900 (51.9)	(<0.001)
	Mean ± SD	3.5 ± 0.76	3.6 ± 0.76	3.3 ± 0.79	−11.15 (<0.001)
Self-rated health	4 or more	2233 (54.3)	1540 (64.7)	693 (39.9)	248.46
	3 or fewer	1881 (45.7)	839 (35.3)	1042 (60.1)	(<0.001)
	Mean ± SD	3.4 ± 0.81	3.6 ± 0.74	3.2 ± 0.88	−17.80 (<0.001)

ADLs = activities of daily living, IADLs = instrumental activities of daily living, SD = standard deviation.

**Table 3:** Probability of driving cessation by health status.

Variable		B	*p*	OR	95% CI
Number of diagnosed chronic diseases (ref. = Less than 2)	2 or more	0.20	0.041	1.22	1.008–1.464
Number of medications (ref. = Less than 5)	5 or more	−0.26	0.222	0.77	0.510–1.169
Diagnosed with chronic disease (ref. = not)	Cerebral infarction	0.45	0.051	1.57	0.998–2.452
	Heart disease	−0.19	0.161	0.83	0.641–1.076
	Diabetes mellitus	0.02	0.852	1.02	0.842–1.232
	Arthritis	0.08	0.571	1.08	0.820–1.434
	Depression	1.29	0.030	3.64	1.133–11.706
	Dementia	0.79	0.252	2.20	0.571–8.450
	Insomnia	−0.07	0.790	0.93	0.558–1.560
	Glaucoma	0.26	0.459	1.30	0.653–2.574
	Presbycusis	−0.27	0.298	0.76	0.459–1.269
	Cancer	−0.33	0.203	0.72	0.436–1.193
	Urinary incontinence	0.58	0.150	1.78	0.813–3.884
	Anemia	0.57	0.177	1.77	0.772–4.072
	Others	−0.38	0.390	0.69	0.291–1.618
ADLs (ref. = independent)	Dependent	0.48	0.071	1.61	0.960–2.701
IADLs (ref. = independent)	Dependent	0.51	0.001	1.67	1.243–2.238
Discomfort (ref. = not)	Vision	0.17	0.048	1.18	1.001–1.396
	Hearing	0.23	0.069	1.26	0.982–1.613
	Mobility	0.23	0.193	1.25	0.892–1.764
Risk of depression (ref. = not)	At risk	0.29	0.015	1.34	1.059–1.691
Cognitive impairment (ref. = not)	Suspected	0.55	<0.001	1.73	1.422–2.116
Quality of sleep (ref. = 4 or more)	3 or fewer	0.17	0.054	1.18	0.997–1.397
Self-rated health (ref. = 4 or more)	3 or fewer	0.19	0.029	1.21	1.020–1.445

χ^2^(*p*) = 1447.90 (<0.001); Nagelkerke R^2^ = 0.399; Hosmer and Lemeshow χ^2^(*p*) = 9.03 (0.340). Adjusted variables: age, educational level, household income quintile, employment status and living arrangement. ADLs = activities of daily living; IADLs = instrumental activities of daily living; OR = odds ratio; CI = confidence interval.

## Data Availability

The data presented in this study are available on [the MicroData Integrated Service (MDIS) website of the National Statistical Office] at [https://mdis.kostat.go.kr/eng/index.do (accessed on 27 May 2025)].
